# Network of depression and anxiety symptoms in Chinese middle-aged and older people and its relationship with family health

**DOI:** 10.1590/1980-220X-REEUSP-2024-0136en

**Published:** 2025-02-07

**Authors:** Shilin Ma, Doudou Huang, Shuangdui Ji, Guangli Mi, Donglian Zheng

**Affiliations:** 1 Ningxia Medical University, School of Nursing, Yinchuan, China.; 2 Ningxia Medical University, General Hospital, Yinchuan, China.; 3 Ningxia Medical University, General Hospital, Nursing Department, Yinchuan, China.

**Keywords:** Anxiety, Depression, Family Health, Middle Aged, Elderly, Ansiedade, Depressão, Saúde da Família, Pessoa de Meia-Idade, Idosos, Ansiedad, Depresión, Salud de la Familia, Persona de Mediana Edad, Ancianos

## Abstract

**Objective:**

To examine the network structure of depression and anxiety symptoms and their association with Family Health among middle-aged and older people in China.

**Method:**

This was a quantitative cross-sectional study, a total of 3,365 middle-aged and older people over 45 years were recruited, comprising 1,748 males and 1,617 females. Data were collected by using Patient Health Questionnaire-9, the Generalized Anxiety Disorder-7, and the Short Form of the Family Health Scale.

**Results:**

The network structure of anxiety and depression symptoms was stable, and “Fatigue” and “Restlessness” were central symptoms and bridge symptoms. “Family, social or emotional health process” and “Family Healthy Lifestyle” exhibited a significant positive correlation, whereas “Family health resources” and “Suicide” demonstrated a significant negative correlation.

**Conclusion:**

“Fatigue” and “Restlessness” are the targeted symptoms for preventing comorbid depression and anxiety symptoms among middle-aged and older adults, and the enhancement of “Family health resources” could be crucial for averting the onset of depression and anxiety symptoms within this demographic group.

## RESUMO

**Objetivo:**

Examinar a estrutura de rede dos sintomas de depressão e ansiedade e sua associação com a Saúde da Família entre pessoas de meia-idade e idosas na China.

**Método:**

Estudo transversal quantitativo, no qual foram recrutadas 3.365 pessoas de meia-idade e idosas com mais de 45 anos, sendo 1.748 homens e 1.617 mulheres. Os dados foram coletados por meio do *Patient Health Questionnaire-9*, do *Generalized Anxiety Disorder-7* e a forma abreviada da *Family Health Scale*.

**Resultados:**

A estrutura de rede dos sintomas de ansiedade e depressão era estável, e “Fadiga” e “Inquietação” eram sintomas centrais e sintomas-ponte. “Processo de saúde familiar, social ou emocional” e “Estilo de vida saudável da família” exibiram uma correlação positiva significativa, enquanto “Recursos de saúde familiar” e “Suicídio” demonstraram uma correlação negativa significativa.

**Conclusão:**

“Fadiga” e “Inquietação” são os sintomas alvos para prevenir sintomas comórbidos de depressão e ansiedade entre adultos de meia-idade e idosos, e o aprimoramento dos “Recursos de saúde da família” pode ser crucial para evitar o aparecimento de sintomas de depressão e ansiedade nesse grupo demográfico.

## RESUMEN

**Objetivo:**

Examinar la estructura de la red de síntomas de depresión y ansiedad y su asociación con la Salud Familiar entre personas de mediana edad y mayores en China.

**Método:**

Se trató de un estudio transversal cuantitativo, en el que se reclutaron 3.365 personas de mediana edad y mayores de 45 años, de los cuales 1.748 eran hombres y 1.617 mujeres. Los datos se recopilaron mediante el Cuestionario de Salud del Paciente-9, el Trastorno de Ansiedad Generalizada-7 y la Forma Abreviada de la Escala de Salud Familiar.

**Resultados:**

La estructura de red de los síntomas de ansiedad y depresión fue estable, y “Fatiga” e “Inquietud” fueron síntomas centrales y síntomas puente. “Proceso de salud familiar, social o emocional” y “Estilo de vida saludable familiar” exhibieron una correlación positiva significativa, mientras que “Recursos de salud familiar” y “Suicidio” demostraron una correlación negativa significativa.

**Conclusión:**

La “Fatiga” y la “Inquietud” son los síntomas objetivo para prevenir los síntomas de depresión y ansiedad comórbidos entre los adultos de mediana edad y mayores, y la mejora de los “Recursos de salud familiar” podría ser crucial para evitar la aparición de síntomas de depresión y ansiedad dentro de este grupo demográfico.

## INTRODUCTION

Since the outbreak of coronavirus disease 2019 (COVID-19) was first reported in China at the end of December 2019, it had spread to over 200 countries and territories by October 2021, with more than 236 million confirmed cases^(1)^. The COVID-19 pandemic has negatively impacted the mental health of vulnerable populations, particularly middle-aged and older individuals, leading to an increased risk of psychiatric issues such as depression and anxiety^(2)^. With strict public health measures and widespread vaccination, the COVID-19 epidemic has been effectively controlled in China since late 2020, although occasional outbreaks due to imported cases from abroad continue to occur^(3)^. Recent studies, however, have revealed that psychiatric issues, such as depression and anxiety, remain prevalent even in the later stages of the pandemic, despite improved containment efforts during this period^(4)^. Consequently, addressing depression and anxiety symptoms among middle-aged and older adults in the latter phases of the COVID-19 pandemic has become a significant focus of both clinical practice and research. Notably, comorbid depression and anxiety are associated with more severe health outcomes compared to either condition alone, leading to increased illness severity, a higher risk of chronicity, and greater functional impairment^(5)^. Therefore, understanding the specific characteristics of comorbid depression and anxiety is crucial for reducing the risk of severe health outcomes in middle-aged and older populations.

A crucial element influencing anxiety and depression among middle-aged and elderly individuals is family health. Relevant studies have demonstrated^(6)^ that depression and anxiety can be influenced by diverse family factors, such as family income, communication, family relationships, and family functionality. As a collective resource, family health emerges from the overall well-being of individual family members, their interactions, capabilities, as well as the physical, social, emotional, economic, and medical resources accessible to the family^(7)^. Hence, the correlation between family health and middle-aged and older people must be considered.

The current research on depression and anxiety in China primarily depends on the latent variable theory, which determines the existence and severity of these mental health issues by evaluating the overall scores on scales or questionnaires. Nevertheless, this method frequently neglects the interaction between depression and anxiety symptoms. Additionally, a direct classification based on the occurrence and severity of symptoms might not take into consideration cases that present similar symptoms due to dissimilar underlying mechanisms.

Network models can make up for this deficiency to some extent by using a web of interacting symptoms to map specific relationships between individual symptoms of a disease^(8)^. Network analysis has been extensively employed in psychopathology in recent years to understand and display patterns associated with mental diseases^(9)^.

To date, no studies on comorbid depressive and anxiety symptoms in Chinese middle-aged and older people and their relationship with family health in the late stage of the COVID-19 pandemic have been published using the network model, which gave us the impetus to conduct this study.

The aim of this study is to explore the network structure of anxiety and depression comorbidity among middle-aged and older individuals in China, utilizing data from the later stages of the COVID-19 pandemic. Furthermore, the study will examine the relationship between family health and both anxiety and depression symptoms. Drawing on insights from the literature review, we propose the following hypotheses: 1. Symptom network structure: Certain symptoms are expected to emerge as core or bridge symptoms within the network, highlighting their critical role in the comorbidity of depression and anxiety. 2. Impact of family health: Specific dimensions of family health, such as emotional support and family functioning, are anticipated to show stronger associations with particular depressive and anxiety symptoms.

## METHOD

### Ethical Consideration

The study adhered to the principles outlined in the Declaration of Helsinki. Ethical approval for all experimental protocols was granted by the Institutional Review Committee of Jinan University, Guangzhou, China (JNUKY-2021-018). The cover page of the questionnaire provided a clear explanation of the study’s purpose and assured participants of anonymity, confidentiality, and the right to refuse participation. Informed consent was obtained from all participants involved in the study.

### Study Design

This study used a quantitative and cross-sectional design. The study report followed the Strengthening the Reporting of Observational Studies in Epidemiology (STROBE) guidelines.

### Study Local

Data for this study were from the Psychology and Behavior Investigation of Chinese Residents (PBICR) survey, a national cross-sectional survey initiated by the Peking University School of Public Health in 2021. Based on the results of the Seventh National Population Census conducted in 2021, a quota sampling method was utilized to select 120 urban residents. The quota attributes included gender, age, and urban-rural distribution, ensuring that the sample characteristics closely aligned with those of the overall population. The survey encompasses 120 cities across 23 provinces, 5 autonomous regions, and 4 municipalities directly under the central government in China.

### Population

In the 2021 PBICR survey, a total of 11,709 questionnaires were collected. After conducting logical checks and eliminating outliers, 11,031 questionnaires were determined to be valid. This study will focus specifically on the age group of 45 years and older. As a result, the final sample size consisted of 3,365 middle-aged and older people following the sorting process. The inclusion criteria for participants in this study were as follows: (1) Participants must be of Chinese nationality residing in the People’s Republic of China; (2) The Chinese resident population includes individuals who have resided in the country for a month or less; (3) They should be able to independently complete the online questionnaire or do so with the assistance of investigators. The exclusion criteria for participants in this study were as follows: (1) Less than 45 years old; (2) Individuals with limited mobility, confusion, or abnormal mental state; (3) Those currently involved in similar research projects.

### Data Collection

This survey was conducted from July 10, 2021 to September 15, 2021, encompassing a total of 120 cities. In each city, at least one investigator or research team was recruited. Each investigator was tasked with collecting between 30 and 90 questionnaires, while each research team was responsible for gathering 100 to 200 questionnaires. Investigators utilized the online questionnaire platform (https://www.wjx.cn/) to distribute questionnaires to the public in their designated areas on a one-on-one basis, with respondents providing their answers by clicking a link. Throughout the questionnaire distribution process, the principles of research design and statistical requirements were adhered to, ensuring control over potential biases in data collection.

### Instruments

#### General Situation Survey Information

The basic demographic information of the older individuals included gender, age rank, education level, Region, Marital status, Per capita monthly household income, and family type (conjugal family, core family, backbone family, and other family), Number of children.

#### The Patient Health Questionnaire-9

The scale utilized in this study consisted of 9 items, with patients rating their discomfort over the past 2 weeks using a Likert level 4 scoring method (0–3). The total score on the scale ranged from 0 to 27, where higher scores indicated more severe depressive symptoms. Scores of 0 to 4 represented no depression, 5 to 9 indicated mild depression, 10 to 14 denoted moderate depression, and 15 to 27 signified severe depression. The Cronbach’s α coefficient for the Patient Health Questionnaire-9 in this study was calculated to be 0.939, and the Kaiser-Meyer-Olkin value was 0.953, which indicates that the reliability and validity of the scale are acceptable.

#### The Generalized Anxiety Disorder-7

Anxiety symptoms were evaluated over a two-week period using a scale consisting of 7 items. A four-point Likert scale (0–3) was employed, resulting in total scores ranging from 0 to 21. Higher scores signified more pronounced anxiety symptoms, with 0–4 categorized as no anxiety, 5–9 as mild anxiety, 10–14 as moderate anxiety, and ≥15 as severe anxiety. The Cronbach’s α coefficient for the scale in this particular study was calculated to be 0.955, and the Kaiser-Meyer-Olkin value was 0.953. The reliability and validity of the scale are acceptable.

#### The Short-Form of Family Health Scale

The scale used to assess family health function consisted of 4 factors and 10 items: internal family emotional communication factor (3 items), family healthy lifestyle factor (2 items), family health resources factor (3 items), and external family social support factor (2 items). Each item was scored on a Likert 5-point scale (1–5 points). The total scale demonstrated a Cronbach’s α coefficient of 0.84, with each dimension showing a Cronbach’s α coefficient ranging from 0.75 to 0.89, which was deemed as indicating acceptable reliability, and the Kaiser-Meyer-Olkin value was 0.702, which was deemed as indicating acceptable validity.

### Data Analysis

Data were organized in a Microsoft Office Excel® spreadsheet through double entry and subsequent validation to control for potential errors in transposing the information. The data were then analyzed using the Statistical Package for the Social Sciences (SPSS®) version 25.0, which facilitated the descriptive statistical analysis of sample characterization. This analysis included the calculation of absolute and relative frequencies, as well as measures of central tendency (mean, median) and variability (standard deviations and interquartile ranges). For numeric variables, the Shapiro-Wilk and Kolmogorov-Smirnov tests were applied to assess adherence to normality, specifically examining the distribution of these data. We performed correlation analysis to assess the relationship between continuous variables. Pearson’s correlation coefficient was used for normally distributed variables, while Spearman’s rank correlation coefficient was used for non-normally distributed variables. The type I error adopted was 5%.

The network analysis was carried out using R 4.3.2 statistical software. Initially, a graphical least absolute shrinkage and selection operator (LASSO) algorithm was employed to establish the network structure. Subsequently, node centrality indices such as strength, tightness, and intermediation were calculated to assess the significance of each symptom within the network structure. The bootnet package was then utilized to examine the invariance of the sequence of nodes on the central index by reducing the sample size in the network structure. The stability of centrality indices was evaluated through the calculation of the correlation stability coefficient. Following the criteria proposed by Epskamp, Borsboom, and Fried, a CS value of 0.70 signifies the maximum acceptable sample reduction level, with a CS coefficient above 0.50 considered acceptable, and not less than 0.25 as the minimum threshold.

## RESULT

### Descriptive Statistics

Patients demographic breakdown is summarized in Table 1. Among the 3,365 participants, 1,748 (51.9%) were male and 1,617 (48.1%) were female. Additionally, 2,153 (63.9%) were middle-aged, while 1,212 (36.1%) were older people. The total scores for depressive symptoms and anxiety symptoms were 14.34 (SD = 5.09) and 10.92 (SD = 4.22), respectively. The total score for the Short-form of Family Health Scale was 38.61 (SD = 6.61). The scores for the four dimensions of the scale were as follows: Family, social, or emotional health processes 12.11 (SD = 2.48), Family healthy lifestyle 8.13 (SD = 1.70), Family health resources 7.67 (SD = 1.63), and Family external social supports 7.67 (SD = 1.63). The means and standard deviations of the psychological scales are presented in Table 2.

**Table 1:** Demographic characteristics of patients (n=3,365) – China, 2021.

Categorical variables	Number (n)	Percentage (%)
**Sex**		
Male	1748	51.9
Female	1617	48.1
**Age group**		
45–65	2153	63.9
>;65	1212	36.1
**Region**		
Urban	2306	68.5
Rural	1059	31.5
**Highest educational level**		
Junior school or below	1446	42.9
Senior school or middle special school	700	20.8
Junior college	512	15.2
Bachelor’s degree or above	707	21
**Marital status**		
Married	2939	87.3
unmarried	120	3.6
Divorced	85	2.5
widowed	221	6.6
**Per capita monthly household income**		
≤3000	1044	31.1
3001–6000	1335	39.7
6001–9000	544	16.1
≥9001	442	9.5
**Family type** ^a^		
Single-parent family	107	3.2
Backbone family	568	16.9
Core family	1592	47.3
Joint family	161	4.8
Conjugal family	757	22.5
other family	162	4.8
**Number of children**		
≥3	520	15.5
0	186	5.5
1	1501	44.6
2	1158	34.4

**Table 2:** Values for psychological variables (n = 3,365) – China, 2021.

Variable	Mean ± SD
The depression symptom	14.34 ± 5.09
The anxiety symptom	10.92 ± 4.22
The total of Family Health	38.61 ± 6.61
Family, social, or emotional health processes	12.11 ± 2.48
Family healthy lifestyle	8.13 ± 1.702
Family health resources	10.73 ± 2.97
Family external social supports	7.67 ± 1.632

### Analysis of Correlation

Table 3 shows the results of correlation analysis between depression and anxiety and the four dimensions of Family Health. Depression symptoms and anxiety symptoms had significant correlations with all four dimensions of family health. As expected, high correlations were observed between the four dimensions of Family Health (r = 0.078–0.884).

**Table 3:** Correlations among Family health, Anxiety, and Depression (n = 3,365) – China, 2021.

	Depression symptom	Anxiety symptom	Family health	Family social/emotional health	Healthy lifestyle at home	Family health resources	Social support outside the family
Depression symptom	1						
Anxiety symptom	0.854**	1					
Family Health	−0.754**	−0.745**	1				
Family social/emotional health	−0.222**	−0.226**	0.339**	1			
Healthy lifestyle at home	−0.210**	−0.215**	0.340**	0.884**	1		
Family Health Resources	−0.314**	−0.286**	0.079**	0.166**	0.165**	1	
Social support outside the family	−0.139**	−0.144**	0.328**	0.666**	0.657**	0.078**	1

Note: **P < 0.01.

### Network Analysis of Depression and Anxiety Symptoms

**Figure 1:**
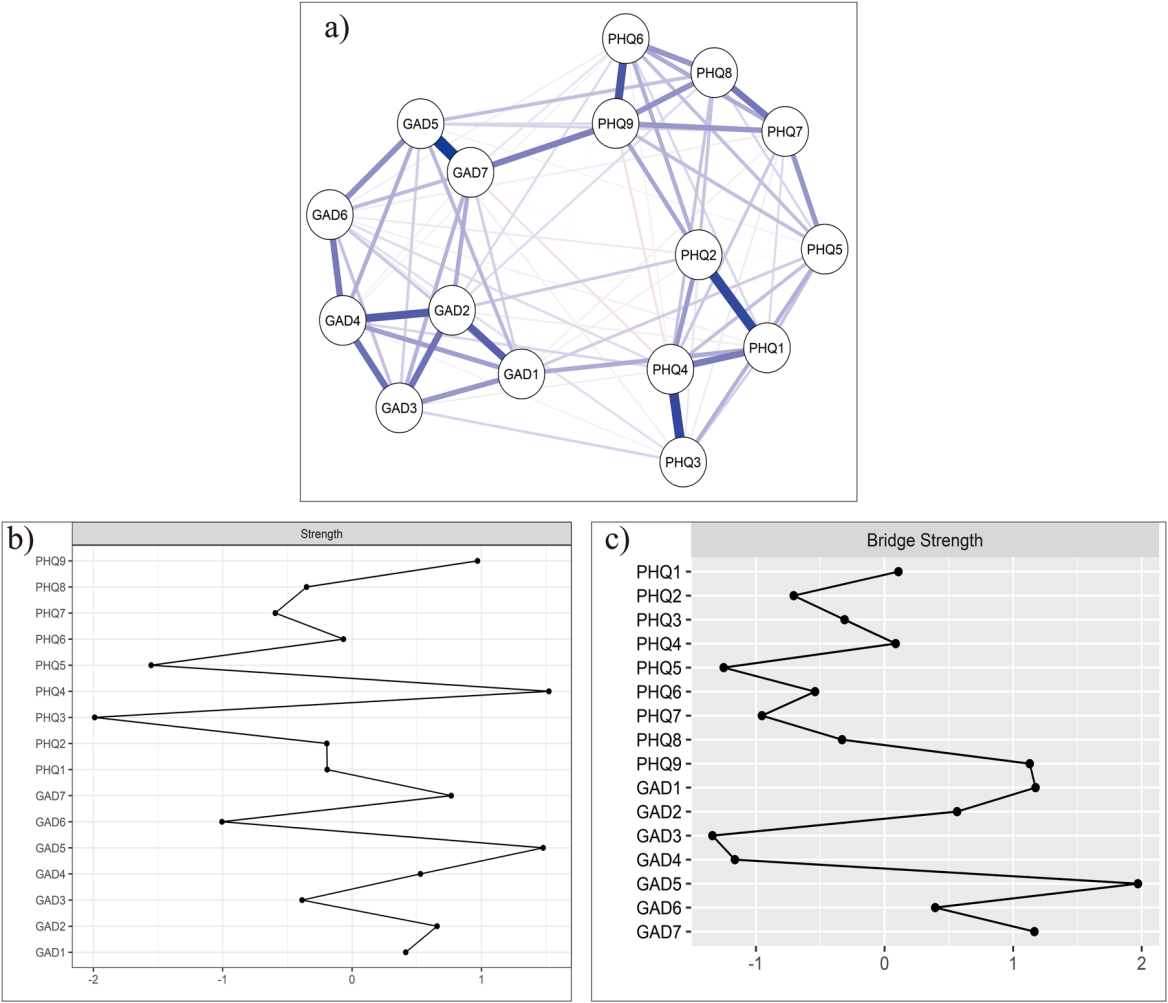
Network model estimation of the depressive-anxiety symptom and Node centrality strength and bridge strength among middle-aged and old people (N = 3,365).

Figure 1(a) shows the network structure of depression symptom and anxiety symptom among middle-aged and old people. The blue line represents positive correlations. The red line represents negative correlations. The edge thickness represents the strength of association between symptom nodes. The network model indicates that the connection between “Restlessness” and “Feeling afraid” was the strongest positive edge in the anxiety community, followed by the edges between “Nervousness” and “Uncontrollable Worry”, and between “Uncontrollable Worry” and “Trouble relaxing”. In the depression community, the edge between “Anhedonia” and “Sad Mood” was the strongest one, followed by the edges between nodes “Sleep difficulty” and “Fatigue” and between nodes “Guilt” and “ Suicide”. For centrality index, the node “Fatigue” had the highest strength centrality in the whole network (Figure 1(b)), indicating that the “Fatigue” symptom was important and influential for understanding the structure of the depression and anxiety network model among middle-aged and old people. For bridge strength index, “Restlessness” was the most key bridge symptom linking depression and anxiety communities (Figure 1(c)).

(A) The blue line represents positive correlations. The red line represents negative correlations. The edge thickness represents the strength of association between symptom nodes. GAD-1:Nervousness; GAD-2: Uncontrollable Worry; GAD-3: Excessive worry; GAD-4: Trouble relaxing; GAD-5:Restlessness; GAD-6: Irritability; GAD-7: Feeling afraid; PHQ-1: Anhedonia; PHQ-2: Sad Mood; PHQ-3: Sleep difficulty; PHQ-4: Fatigue; PHQ-5: Appetite; PHQ-6: Guilt; PHQ-7: Concentration; PHQ-8: Motor; PHQ-9: Suicide. (B) Node centrality strength and bridge strength in the depressive-anxiety symptom network.

### Network Stability of Depression and Anxiety Symptoms

**Figure 2:**
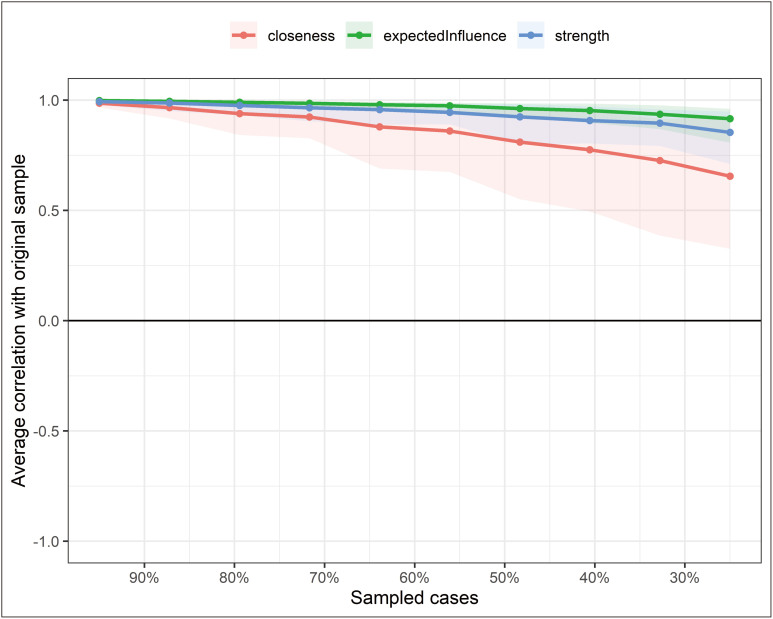
The stability of centrality and bridge centrality using case-dropping bootstrap (N = 3,365).

Figure 2 shows a stability analysis of the network and found that the centrality of strength had an excellent level of stability (i.e., CS-coefficient = 0.75), which indicates that 75 % of the samples could be eliminated without significant changes in the network structure.

### Network Structure of Family Health Dimensions and Depression and Anxiety Symptoms

**Figure 3:**
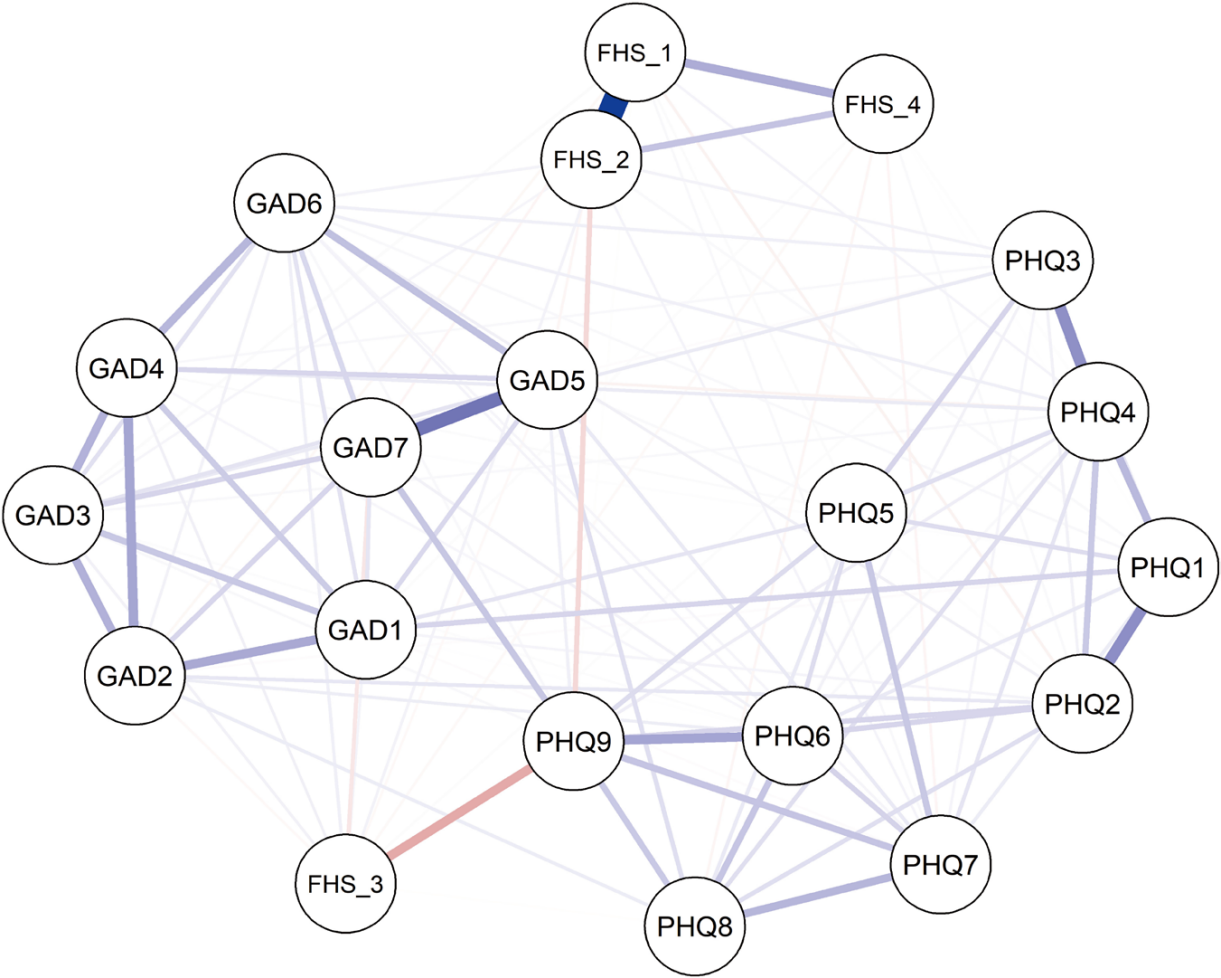
Family Health and Depressive Symptoms and Anxiety Symptoms Network (N = 3,365).

Figure 3 shows the network structure of family health dimensions and depression and anxiety symptoms. In the family health section, “Family healthy lifestyle” showed strongest positive correlations with “Family, social, or emotional health processes”. Overall, Family health was negatively associated with depressive symptoms. “Family health resources” showed a distinct negative correlation with “Suicide”. For bridge strength index, “Family health resources” and “Suicide” were the most key bridge symptoms linking depression and anxiety symptoms communities.

The blue line represents positive correlations. The red line represents negative correlations. The edge thickness represents the strength of association between symptom nodes. FHS_1: Family, social, or emotional health processes; FHS_2: Family healthy lifestyle; FHS_3: Family health resources; FHS_4: Family external social supports.

## DISCUSSION

In this study, we utilized data from the Psychology and Behavior Investigation of Chinese Residents during the late stage of COVID-19 to explore the network structure of comorbid depression and anxiety symptoms among middle-aged and older Chinese individuals, as well as to investigate its association with family health. Our findings indicate that the comorbidity of anxiety and depression symptoms demonstrates a stable network structure, with individual symptoms being interrelated. Furthermore, family health was found to be negatively correlated with the overall symptoms of depression and anxiety. In the present study, “Fatigue” was the most central symptom in the network of depression and anxiety. In addition, “Restlessness” was the key bridge symptom linking depression symptom and anxiety symptom in this study. Thus, these symptoms are important and influential in understanding the structure of network models of depression symptom and anxiety symptom in middle-aged and older adults. Our findings indicate that family health is negatively associated with symptoms of depression and anxiety. We verified that the factor “family, social, or emotional health processes”, which constitutes family health, is strongly correlated with “Family healthy lifestyle”. Additionally, “Family health resources” exhibited a significant negative correlation with “Suicide” in relation to depressive symptoms.

In the depression symptom and anxiety symptom network, “Restlessness” was the central and hallmark symptom of Chinese middle-aged and older people. This finding aligns with the results of a previous study conducted on residents of Macau, China^(10)^. However, previous studies found that other depressive symptoms, such as “Sad mood” and “Too much worry”, were also the most central symptoms among Filipino domestic workers^(11)^. These inconsistent findings indicate the discrepancy of central symptoms among different study samples and different study periods. In the network theory, central symptoms play important roles in maintaining the psychopathology network, hence treating those symptoms could help to ameliorate the relevant psychopathology^(5)^. The importance of “Fatigue” in the network structure in terms of being highest in strength may be due to somatization of distress, which is common among Asian populations^(12)^. Previous studies have suggested^(13)^ that both men and women are more likely to experience fatigue as they age. Furthermore, a sedentary lifestyle may contribute to diminished physical strength and heightened fatigue among middle-aged and older people^(14)^. Consequently, “Fatigue” is not only a prominent symptom of mental illness but also a common daily experience. In addition, a previous study has indicated that fatigue is a common complaint in both the general population and patients with various disorders^(15)^. Furthermore, “Fatigue” is a common symptom of depression, affecting more than 90% of patients, and is recognized as the most frequent symptom of insomnia^(16)^. This prevalence and association could explain the strong association between “Fatigue” and other symptoms, especially “Difficulty sleeping”. Thus, addressing “Fatigue” could serve as an effective target for intervention strategies. “Restlessness” as a bridge symptom in the network has also been found in another study^(9)^. In general, these findings suggest that bridge symptoms in different mental disorder communities contribute to psychiatric comorbidity and warrant attention as targets in treatment studies designed to identify particular symptoms most critical for decreasing risk of contagion between psychiatric syndromes within network models^(8)^.

In this network, we found that the strongest edges were all within particular mental disorder communities rather than between different disorders, consistent with the findings of a previous network study^(17)^. In this network analysis, we observed that the strongest edges were predominantly located within specific mental disorder communities, rather than between different disorders. This observation aligns with the findings of prior network research. Notably, the strongest edges identified across the network were between “Restlessness” and “Feelings of fear”, corroborating earlier study findings^(18)^. The second strongest association identified in our sample was between “Anhedonia” and “Sad Mood”, representing a novel link that has not been previously documented. This association may be specific to the current sample within the context of the COVID-19 pandemic. “Anhedonia” is characterized by the inability to experience pleasure from rewarding or enjoyable activities and “Sad mood” involves feelings of sadness, unhappiness, or dejection^(19)^. During the late stage of the pandemic, uncertainty remained due to the rapid spread of SARS-CoV-2 Delta variant globally, necessitating the continuation of strict public health measures^(20)^. In order to protect public health, governments of several countries have been forced to take protective measures. These actions have included closing some cities, shops, schools, and declaring quarantines, and lockdown to impose social distancing. Lockdown is one of the oldest and most effective tools for controlling outbreaks of communicable diseases^(21)^. While, on the one hand, staying at home is a safety measure, it may have unwanted negative consequences, such as less physical activity, increased consumption of certain foods, and increased anxiety levels, impacting general and mental health and increasing “Anhedonia” and “Sad Mood”^(22)^.

We also found that family health was negatively correlated with depression and anxiety symptoms, that is, the higher the level of family health, the lower the risk of anxiety and depression symptoms in middle-aged and old people. In the network of family health and depression-anxiety symptoms, we identified that “Family, social, or emotional health processes”, “Family healthy lifestyle”, “Family health resources”, and “Suicide” were the most influential nodes within the network structure. This indicates that these symptoms are most likely to trigger or sustain family relationships and depressive symptoms. In the present study, it is evident that “Family, social, or emotional health processes” is closely related to “Family healthy lifestyle”, aligning with the previous findings by Fei Wang et al.^(23)^. A concept analysis revealed that family, social or emotional health processes and family healthy lifestyle are mutually influenced, both have a two-way effect, and both jointly affect the health level of the whole family^(24)^. In the present study, “Family health resources” showed a clear negative association with “suicide”. A previous study found^(25)^ that poor “Family health resources” increase the likelihood of mental health problems, physical decline, and premature death. In addition, “Family health resources” is one of the dimensions that constitute family health, and it is also a bridge symptom that connects the network structure of family health and depression and anxiety in middle-aged and older people in this study. The improvement of “Family health resources” can help to improve family health, promote mental health of middle-aged and older people, and prevent the occurrence of depression and anxiety symptoms.

The strengths of this study include a large sample size and the application of network methods to visualize the relationships between comorbid depression and anxiety symptoms and family health among middle-aged and older individuals in China. However, several limitations should be acknowledged. Firstly, as this is a cross-sectional study, it cannot establish causality regarding changes in symptoms over time; thus, future longitudinal studies are warranted. Secondly, depression symptoms and anxiety symptoms were assessed using self-reported questionnaires, which may introduce recall bias. Lastly, in accordance with the reviewer’s observations, it is important to note that this study was conducted during a pandemic. Major public health events can significantly impact mental health in middle-aged and older adults, potentially resulting in increased incidence or severity of anxiety and depression. These implications for the estimated network structure warrant further investigation in future research.

## CONCLUSION

In summary, based on the network perspective, this study constructed the network structure of depression-anxiety symptoms in Chinese middle-aged and older people, and the effect path of family health on depression-anxiety comorbidity. To a certain extent, this study increases the understanding of depressionanxiety comorbidity and can provide theoretical guidance and scientific basis for targeted intervention treatment..
